# Fostering Regeneration and Functional Improvement in the Injured Spinal Cord by a Novel, Stem Cell Secretome-Based Drug Delivery Method

**DOI:** 10.3390/pharmaceutics18060658

**Published:** 2026-05-27

**Authors:** Zoltán Fekécs, Krisztián Pajer, Tamás Bellák, Dénes Török, Zsuzsanna Táncos, Csilla Nemes, László Gál, Julianna Kobolák, András Dinnyés, Antal Nógrádi

**Affiliations:** 1 Department of Anatomy, Histology and Embryology, Albert Szent-Györgyi Medical School, University of Szeged, 6724 Szeged, Hungary; 2 BioTalentum Ltd., 2100 Gödöllő, Hungary; 3 Department of Physiology and Animal Health, Institute of Physiology and Animal Nutrition, Hungarian University of Agriculture and Life Sciences, 2100 Gödöllő, Hungary

**Keywords:** lesion-induced secretome, spinal cord injury, functional recovery, delivery routes

## Abstract

**Background/Objectives**: Spinal cord injury (SCI) causes permanent tissue damage and severely limits functional recovery. We have previously shown that grafted neuroectodermal stem cells (NE-GFP-4C) promote regeneration and motor improvement of the injured cord through the release of specific factors, including Glial cell line-Derived Neurotrophic Factor, interleukin-6, interleukin-10 and Macrophage Inflammatory Protein-1α. Here, we examined whether various delivery routes for these factors could reproduce the effects of stem cell transplantation. **Methods**: Specifically, these factors were administered either in soluble form via osmotic pumps or via genetically modified fibroblasts. NE-GFP-4C cells served as a positive control, while saline and non-transfected fibroblasts were used as controls. Functional and morphological analyses were conducted to assess the therapeutic impact of the secretome treatment. **Results**: Both secretome-treated groups and stem-cell-grafted animals showed significantly improved motor outcomes, reduced lesion volumes, enhanced axonal regeneration and decreased astrocytic and microglial activation. **Conclusions**: These findings suggest that secretome-based delivery of selected factors is a minimally invasive and effective therapeutic strategy that can mimic the regenerative benefits of stem cell transplantation in SCI.

## 1. Introduction

Damage to the spinal cord leads to a loss of autonomic, sensory and motor function distal to the injury, and patients often become completely immobilized [1]. The primary damage is caused by the triggering insult (mechanical impact), which includes destruction of axons and cells (both neurons and glial cells) and loss of integrity of the blood–brain barrier [2]. Edema and hematoma contribute significantly to the subsequent secondary tissue loss, and the damage signals initiate an inflammatory cascade [2,3]. The process begins with the invasion of the lesion site by neutrophil granulocytes, followed by the appearance of T-lymphocytes and macrophages as members of the adaptive immune system [4]. As a result of these events, the inflammatory process is augmented, and proinflammatory cytokines are released from the activated astroglia and macrophages. Simultaneously, reactive oxygen and nitrogen radicals appear and aggravate the primary injury, causing further cell death leading to secondary damage.

Stem-cell-based therapy represents a promising experimental approach for the treatment of spinal cord injury (SCI). Numerous studies have demonstrated both functional improvement and morphological recovery following transplantation of various stem cell types into the injured spinal cord [3,5,6,7]. While transplanted cells have been shown to integrate into the host tissue and differentiate into cell types such as astrocytes and oligodendrocytes [8,9], accumulating evidence suggests that their therapeutic efficacy is driven predominantly by paracrine secretory mechanisms (secretome). In particular, the secretome produced by grafted stem cells plays a pivotal role in modulating the injury microenvironment, thereby creating conditions that are more permissive for regenerating neurons [10,11,12]. The beneficial effects of the secretome have been largely attributed to the secretion of anti-inflammatory mediators, cytokines and growth factors, which contribute to tissue repair following traumatic injuries of the central nervous system (CNS) [13,14,15]. These findings have led to the development of a novel therapeutic paradigm that seeks to harness the regenerative potential of stem-cell-derived secretomes, thereby circumventing the limitations associated with cell transplantation [10,16,17].

In our earlier studies we provided evidence that grafting a neuroectodermal stem cell line (NE-GFP-4C cell line, ATCC No: CRL-2926) into the injured cord resulted in significant functional recovery supported by extensive axonal regeneration and neuroprotection [5,18]. The grafted stem cells minimally contributed to the exogenous cellular replacement, but produced a so-called lesion-induced secretome for 5–7 days after transplantation. This secretome consisted of Glial cell line-Derived Neurotrophic Factor (GDNF), interleukin-6 (IL-6), interleukin-10 (IL-10) and Macrophage Inflammatory Protein-1 Alpha (MIP-1 alpha) [5,6,18]. Functional blocking of the secretome with neutralizing antibodies completely abolished the effects of the grafted cells, proving that these factors played a pivotal role in the significant functional recovery and morphological improvement following contusion injury [5,13]. In this study, we investigated whether various drug delivery methods specialized for a lesion-induced secretome of the clonal neuroectodermal stem cell line NE-GFP-4C can produce the same morphological and functional improvement after a spinal cord contusion injury as grafting of stem cells directly into the lesion cavity.

## 2. Materials and Methods

### 2.1. Study Approval

The experiments were performed with the approval of National Food Chain Safety Office of Hungary (NÉBIH) under protocol number VII./136/2013 and I./2120/2019. All the procedures were performed according to the Helsinki Declaration on Animal Rights. Animals were given food and water ad libitum.

### 2.2. Sex as a Biological Variable

Only female Sprague–Dawley rats were used in this study. The use of females was based on their lower and more stable body weight gain compared to males, which is critical for the accuracy of video-based gait and locomotion pattern analysis. Although a correction method was applied to compensate for variations in weight, step duration and step length, minimizing body weight variability at the experimental design level was considered essential to ensure the robustness and consistency of the motion tracking system.

### 2.3. Image Processing and Statistical Analysis

Graphs and diagrams were created by Microsoft Office Pro Plus 2016 (Microsoft Corporation, Redmond, WA, USA, http://www.office.com, accessed on 10 December 2025). Graphs, diagrams and representative images were further processed using the GNU Image Manipulation Program (GIMP 2.10.0, http://www.gimp.org, accessed on 10 October 2025). No manipulation of the figures was performed other than adjusting the contrast of the images.

The repeated measures two-way ANOVA was applied to compare BBB scores throughout the experimental groups (SPSS version 24.0, IBM Corporation, Armonk, NY, USA). Least Significant Difference (LSD) post hoc analysis was used for multiple comparisons (RStudio Version 1.1.423–© 2009–2018 RStudio, Inc., Boston, MA, USA). At the end of survival time, the treated groups (Groups 1, 3, 4 and 6) were compared to their control groups (Groups 2, 5 and 7). Therefore, the analysis of locomotion pattern, quantitative immunohistochemistry, number of neurons, cavity length and remyelination for each group were analyzed using the Student’s *t*-test (RStudio). The extent of tissue-sparing data was compared using repeated measures ANOVA with LSD post-hoc test (SPSS). One-way ANOVA with LSD post-hoc test (SPSS) was used to compare Groups 1, 3, 4 and 6. Data are presented as mean ± Standard Error of Mean (SEM), and * *p* < 0.05 ** *p* < 0.01 and *** *p* < 0.001 were considered to be significant.

### 2.4. Maintenance of NE-GFP-4C Stem Cells

The NE-GFP-4C cell line (available from ATCC No. CRL-2926), derived from the forebrain vesicles of p53-deficient 9-day-old mouse embryos was used for transplantation. This immortalized cell line expresses Green Fluorescent Protein (GFP), displays several neural stem cell properties and is able to generate neurons and astroglial cells upon induction with all-trans-retinoic acid [19,20].

The maintenance of NE-GFP-4C cells was based on previously described methods [5,6]. NE-GFP-4C cells were maintained on Nunclon Petri dishes (Invitrogen, Thermo Fisher Scientific, Waltham, MA, USA) in High glucose Dulbecco Modified Essential Medium (H-DMEM, Sigma-Aldrich, St. Louis, MO, USA) supplemented with 10% fetal bovine serum (FBS, Gibco, Thermo Fisher Scientific, Waltham, MA, USA) at 37 °C and in 5% CO_2_. Cells were cultured in a humidified atmosphere and passaged twice every week using a 0.1% trypsin solution. All cell cultures underwent at least two, but no more than five passages before transplantation.

### 2.5. Isolation of Rat Embryonic Fibroblasts

Embryonic fibroblast preparation was based on previously described methods [21]. Embryos (E13,5) were removed from pregnant Sprague–Dawley rats and collected in phosphate-buffered saline (PBS). Embryos were then transferred to a 10 cm dish filled with PBS and the heads and viscera of the embryos were removed. The remaining tissues were cut to small pieces and trypsinized (0.1% trypsin solution) in a 37 °C incubator for 20 min, using a magnetic stirrer. Trypsin digestion of the suspension was stopped with HDMEM (Sigma-Aldrich, St. Louis, MO, USA) supplemented with 10% FBS (Gibco, Thermo Fisher Scientific, Waltham, MA, USA). The cell suspension was filtered with metal Sigmathen 100 and 70 µm filters (Merck-Millipore, Merck KGaA, Darmstadt, Germany). Finally, the cells were transferred to T75 flasks (Sarstedt, Nümbrecht, Germany) and kept in a CO_2_ thermostat at 37 °C. The medium was changed twice a week.

### 2.6. Transfection of Embryonic Fibroblasts

The 2A peptide-linked polycistronic vector carrying four human factors (IL-10, IL-6, GDNF and MIP-1α) and the eGFP reporter gene was cloned into a polycistronic pVAX-based Sleeping Beauty transposon system vector backbone (pVAX-SB). The correct assembly was validated by restriction enzymatic digestion and sequencing. Successful protein expression was proven by GFP expression, and specific immunostainings verified the overexpression of the human proteins.

The 4th passage of embryonic fibroblasts was transfected with the X-tremeGene DNA transfection reagent [Cat. No: 6366244001; Roche, Budapest, Hungary; ratio of transfection reagent (µL) to the amount of DNA (µg) = 4:1, with 2 µg of DNA used]. The cells were collected one day later. Higher density samples (65,000 cells/cm^2^) were used for ELISA measurements; lower density (13,000 cells/cm^2^) samples were fixed with 4% buffered paraformaldehyde and processed for immunohistochemistry.

### 2.7. Spinal Cord Contusion Model

Altogether, 105 female Sprague–Dawley rats (200–220 g body weight) were used. All rats were anesthetized with intraperitoneal injections of Ketasol^®^ (dosage: 100 mg/kg, Ketamine hydrochloride 100 mg/mL, Dr. E. Graeub AG, Bern, Switzerland) and Rompun^®^ (dosage: 10 mg/kg Xylazine-Hydrochloride, γ-Hydroxybenzoacidmethylesterate, 2%, Bayer, Leverkusen, Germany). Core body temperature was monitored and maintained at 37 ± 0.5 °C with a heating pad (Supertech Ltd., Pécs, Hungary) until recovery. The lamina of the thoracic 10 vertebra (Th10) was removed and the dura mater was left intact. A contusion injury was performed through the use of a custom-made spinal cord impactor (developed in our laboratory), applying 150 kdyn force. The integrity of the dura mater was checked and the wound was closed in anatomical layers. Postoperative management was uniform for all rats in the study. Subcutaneous injections of 10 mL Ringer’s solution and antibiotics (Peni-Strepto^®^, 50,000 IU, Virbac Lab., Carros, France) as well as analgesics (Metacam; 0.5 mg/kg body weight, Boehringer Ingelheim Vetmedica, St. Joseph, MO, USA) were administered promptly after surgery and once daily for a further three days. The bladders of the operated rats were manually expressed three times a day in the first week or until reflexive function was observed. Rats were housed under simulated daylight conditions with alternating 12 h light–dark cycles. Standard rat food and water were provided ad libitum and the animals were checked daily for signs of infection or dehydration.

### 2.8. Transplantation of NE-GFP-4C Stem Cells, Transfected and Untransfected Fibroblasts

To set up a positive control group, NE-GFP-4C neuroectodermal stem cells were grafted seven days after the injury into the lesion cavity (Group 1). Under deep ketamine–xylazine anesthesia, a small hole was created on the dorsal wall of the lesion cavity in the exposed spinal cord and 5 × 10^5^ cells were grafted into the cavity. Similarly, in Groups 6 and 7, transfected and untransfected fetal fibroblasts (5 × 10^5^ cells in each group) were grafted, respectively.

### 2.9. Implantation of Osmotic Pumps

Osmotic pumps (2ML4, Alzet, Cupertino, CA, USA, 100 µL volume for 14 d) were placed subcutaneously in the back area and equipped with intraspinally applicable flexible silicone tubes (0.1 mm internal diameter; Aran Packaging, Kibbutz Nachshon, Israel) in order to deliver the factors directly to the lesion site [5,6]. First, the most effective dose of the factors was investigated with an increasing dose series of IL-10 (7.5, 15, 22.5 and 45 ng/mL [*n* = 3 each, Supplementary Figure S1]. For the settings of our previous osmotic pump applications and dose-response results, see Pajer et al. 2014; 2019 [5,6]). Our results showed that the 22.5 ng/mL dose was the most appropriate for further application (Supplementary Figure S1). The administration of the factors (22.5 ng/mL each) was initiated seven days after contusion injury and the treatment lasted for two weeks (Groups 2 and 3). Animals in the control group (Group 4) were set up the same way, but received only saline infusions via the osmotic pumps.

### 2.10. Experimental Groups

The following experimental groups were established in this study:

Group 1. (NE-GFP-4C): Transplantation of NE-GFP-4C neuroectodermal stem cells (5 × 10^5^) into the lesion cavity (positive controls, *n* = 13);

Group 2. (SCI): Animals received a single injection of DMEM (no osmotic pumps, native solvent negative control group, *n* = 13);

Group 3. (four factors): Treatment with a continuous infusion of GDNF, IL6, IL-10 and MIP-1alpha via an osmotic pump (*n* = 13);

Group 4. (IL-10 only): Animals were treated with a continuous infusion of IL-10 only via an osmotic pump (*n* = 13);

Group 5. (saline control): Animals in this group received saline for 2 weeks via osmotic pumps (control saline pump experiment of Groups 3 and 4, *n* = 13);

Group 6. (transfected fibroblast): Animals received fetal fibroblast grafts (5 × 10^5^ cells) transfected with the pVAX-SB construct expressing the four factors (*n* = 15);

Group 7. (untransfected fibroblast): Animals in this group received untransfected fetal fibroblast grafts (5 × 10^5^ cells), control of Group 6 (*n* = 13).

### 2.11. In Vivo Validation of the Four-Factor Expression by Transfected Fibroblast

The animals grafted with transfected fibroblasts (Group 6) were sacrificed and perfused transcardially (*n* = 2) with 4% phosphate-buffered paraformaldehyde (PFA) one week after transplantation. The spinal cords of the animals were collected and placed into 4% buffered PFA for one day. After cryoprotection in 30% sucrose in PBS, the samples were embedded in Shandon Cryomatrix gel (Thermo Fisher Scientific, Waltham, MA, USA), and sagittal sections (16 μm thick) were cut on a cryostat (CM 1950, Leica, Nussloch, Germany). Immunohistochemistry was performed to validate the expression of the four factors.

### 2.12. Functional Investigations

The animals were monitored by open-field test (BBB test) initially on the third day after the injury and then weekly for 10 weeks (*n* = 5 in each group) [22]. The rats were briefly assessed by two independent observers in an open field (150 × 100 cm) for 4 min at similar times of the day for each testing.

The detailed locomotor pattern of the rats was evaluated through the use of a video-based kinematic analysis on week 10 after the injury as well (for details see Török D. 2022, [23]). For both hind limbs, eight different parameters were measured to obtain detailed information about the functional recovery. From the lateral view, the following 5 parameters were analyzed: toe-off angle (TOA), knee flexion (KF), ankle flexion (AF), knee lifting (KL) and ankle lifting (AL). The lateral placing parameter (LP) was measured from the ventral view. The metatarsus-surface angle (MSA) and the tibia-surface angle (TSA) were observed from the rear view [23].

### 2.13. Retrograde Labeling

Retrograde labeling of the regenerating and surviving (spared) axons of neurons was performed one week before sacrificing the animals (*n* = 5 each in groups). A laminectomy of the L1 vertebra was carried out and the spinal cord was exposed. After a right hemisection of the L3 spinal segment, Fast Blue crystals (Illing Plastics GmbH, Breuberg, Germany) were placed into the hemisection gap, and one week was allowed for transport after wound closure. Retrogradely labeled neurons were mapped and counted in the motor and sensory cortex, brainstem, and spinal cord above the level of the contusion injury.

### 2.14. Anterograde Labeling

Nine weeks after surgery, four animals per group were deeply anesthetized as described above and processed for anterograde labeling of the corticospinal tract. The skull above the right motor cortex was opened, a dura flap was created and the cortex was exposed. Biotinylated dextran amine (BDA, 10 kDa, Molecular Probes 2.5 mg/mL, Sigma-Aldrich, St. Louis, MO, USA) was injected at 6 injection points into the motor cortex through the use of a fine glass micropipette joined to a Hamilton syringe (Hamilton Bonaduz AG, Bonaduz, Switzerland). The animals were re-anesthetized and perfused transcardially 14 days after the labeling. The anterogradely labeled corticospinal axons were mapped around the injury site.

### 2.15. Tissue Processing

The animals were anesthetized as described above and perfused transcardially at the end of the survival period with PFA. For immuno- and lectin histochemical analysis, the spinal cord, brainstem, and the brain were removed and postfixed in PFA for one day. The fixed tissues were cryoprotected in 30% sucrose in PBS containing 0.01% sodium-azide at 4 °C until being embedded in Shandon Cryomatrix gel (Thermo Fisher Scientific, Waltham, MA, USA). Parallel or serial transverse (25 μm thick) and longitudinal (16 μm thick) sections were cut on a cryostat (CM 1950, Leica, Nussloch, Germany) and mounted onto gelatine-coated glass slides. The extent of remyelination, the length of cavity and the extent of spared tissue were determined from paraffin-embedded tissues. In this case, the spinal cord samples taken from the injury site were placed into 2% osmium-tetroxide for 2 h. After thorough washing in PBS, the samples were embedded into solid paraffin, and serial transverse (5 μm thick) sections were cut with a rotating microtome (Thermo Scientific, Waltham, MA, USA). Every fifth section was mounted onto glass slides and used.

### 2.16. Immuno- and Lectin Histochemistry

Nonspecific binding sites were subsequently blocked with 3% normal donkey or goat serum. Primary antibodies were used as follows: rabbit anti PKC-γ (1:100, ab187746, Abcam, Cambridge, UK), rabbit anti-GFAP (1:400, 18-0063, Thermo Fisher Scientific, Waltham, MA, USA), mouse anti-CS-56 (1:200, C8035, Sigma-Aldrich, St. Louis, MO, USA), rabbit anti-GDNF (1:200, ab18956, Abcam, Cambridge, UK), mouse anti-IL-6 (1:250, ab9324, Abcam, Cambridge, UK), rabbit anti-IL-10 (1:150, E92171, Enogene, Nanjing, China), and rabbit anti-MIP-1-alpha (1:200, ab9781, Abcam, Cambridge, UK). The immune reaction was completed by Alexa Fluor 488 goat anti-rabbit (1:600, A11008, Thermo Fisher Scientific, Waltham, MA, USA), Alexa Fluor 594 donkey anti-mouse (1:600, A21203, Thermo Fisher Scientific, Waltham, MA, USA), Streptavidin Alexa Fluor 546 Conjugate (1:400, S-11225, Thermo Fischer Scientific, Waltham, MA, USA), and Alexa Fluor 546 goat anti-rabbit (1:400, A11010, Thermo Fischer Scientific, Waltham, MA, USA).

For the labeling of microglia, biotinylated Griffonia Simplicifolia isolectin B4 (GSA-IB4, 1:200, B1205, Vector Laboratories, Newark, CA, USA) was used. Sections were treated with DAPI [2-(4-carbamimidoylphenyl)-1H-indole-6-carboximidamide; 1:2000, Biotium, Fremont, CA, USA, 40011] for staining the cell nuclei. Fluorescent images were obtained by using a BX-50 epifluorescent microscope equipped with a DP-74 digital camera (Olympus, Tokyo, Japan).

### 2.17. Quantitative Assessment of Retrograde Tracing

To determine the number of retrogradely labeled neurons, a previously described method was used [24]. Serial transverse sections (25 μm thick) were taken from the T5, T2, C6, and C2 spinal segments, and every tenth coronal section (25 μm thick) was used from the brainstem and the cerebral cortex. To avoid double counting of the same neuron in the spinal cord segments, the FB-labeled neurons were mapped and their location was compared with that of the labeled neurons in the adjacent sections [25].

### 2.18. Quantitative Determination of PKCγ-Positive Area in the Corticospinal Tract

The sparing/regeneration of corticospinal tract was determined according to the previously described methods [26]. Serial cross-sections (25 μm thick) were taken proximally and caudally from the site of contusion injury (2 mm rostral and caudal to the tip of injury). To assess the size of PKCγ-positive area in the posterior funiculi of spinal cord segments of control and treated groups (*n* = 4 in each group), photographs were taken from the sections in each rat. The area of PKCγ immuno-reactivity was determined through the use of the ImageJ Software (ImageJ 1.51j8, Wayne Rasband, National Institutes of Health, Bethesda, MD, USA, https://imagej.nih.gov/ij, accessed on 15 August 2025) and expressed in mm^2^.

### 2.19. Quantification of GFAP, GSA-IB4 and CS56 Densities Ten Weeks After the Injury

To assess the densities of GFAP-positive astrocytes, GSA-IB4-positive microglia/macrophages and CS56-positive chondroitin sulphate deposits in the spinal cords of control and treated animals (*n* = 4 in each group), photographs were taken from sagittal sections (150 µm apart) of each spinal cord. The samples contained the lesion cavity and 2 mm long parts of the spinal cord related both to the rostral and caudal ends of the cavity. The relative densities of GFAP and CS56 immunoreactivities and that of GSA-IB4 lectin reactivity were determined through the use of the ImageJ Software (ImageJ 1.51j8, Wayne Rasband, National Institutes of Health, Bethesda, MD, USA, https://imagej.nih.gov/ij, accessed on 15 August 2025). To correct for interanimal variations in the immunolabeling efficiency, the density of the immunolabeled tissue was normalized to the uninjured area of the same section 4 mm rostral to the end of the injured area. Data were expressed as an n-fold increase in immuno-density normalized to the uninjured value.

### 2.20. Quantification of Cavity Length and Tissue Sparing

To determine the cavity length and the tissue sparing in the spinal cords of control and treated animals (*n* = 4 in each group), photographs were taken from every fifth osmium-tetroxide-treated transverse section containing the lesion cavity and 1 mm long parts of the spinal cord related both to the rostral and caudal ends of the cavity. The boundary between the intact tissue and the lesion cavity consisting of small cysts was determined. Cavity length was computed using the following equation: cavity length (mm) = section thickness (μm) × number of sections.

The extent of spared tissue was determined as follows: the number of pixels in the reference area (1 mm^2^) and the area of the entire spinal cord segments was calculated using the NIH ImageJ analysis software. The number of pixels of the spared tissue was measured at the epicenter (0) and 0.3, 0.6, 0.9, and 1.2 mm rostrally and caudally from it. The amount of spared tissue was normalized to the entire spinal cord area. The proportion of spared tissue in the injured animals was given in mm^2^.

### 2.21. Quantitative Assessment of Myelinated Fibers

The number of myelinated axons was determined according to a previously described method [27]. Images were taken from the osmium-tetroxide-treated transverse sections (5 µm thick) containing the center of the lesion site. The number of axons with intact myelin sheaths was counted through the use of the NIH ImageJ Software in 6 randomly selected 100 µm × 100 µm white matter areas of ventral and lateral funiculus. The numbers were normalized to the total area of spared white matter.

## 3. Results

### 3.1. Analysis of Gene Expression Induced by the Polycistronic pVAX-SB Vector

**Figure 1:**
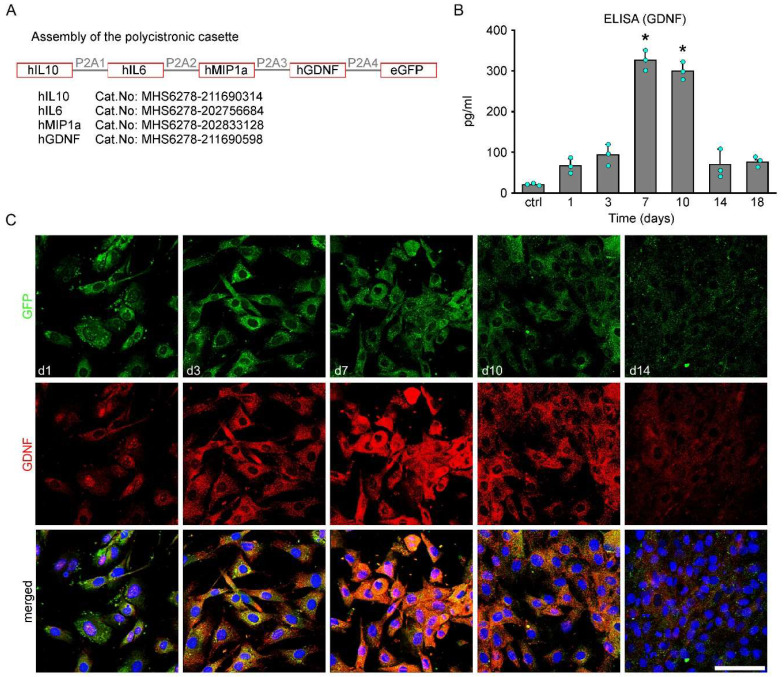
**Expression of GDNF by transfected embryonic fibroblasts in vitro.** (**A**) Schematic representation of the polycistronic pVAX-SB vector encoding the factors of the lesion-induced secretome and GFP. (**B**) Time course of GDNF levels produced by transfected fibroblasts in vitro, detected by ELISA. The peak values of GDNF were secreted on Days 7 and 10. The GDNF values at these two days were significantly different (asterisk) compared with that of Day 1. (**C**) Confocal images show temporal expression of GFP and GDNF colocalized with the nuclear fluorescent marker DAPI (blue) in embryonic fibroblasts transfected by polycistronic pVAX-SB cassette. Data are presented as mean ± SEM. * *p* < 0.05; Scale bar: 20 µm in (**C**).

To verify the biological action of the pVAX-SB vector, in vitro experiments were first performed to determine the amount and the time period of secretion of factors. The four genes were coupled to a common promoter in the pVAX-SB vector (Figure 1A, Supplementary Figure S2). Embryonic rat fibroblasts transfected with the vector expressed all factors simultaneously for approximately 12 days. ELISA measurement of GDNF expression from transfected fibroblasts showed a peak expression on Days 7 to 10 after transfection (Figure 1B). Moreover, the initial increasing and then decreasing gene expression pattern of GDNF was verified immunohistochemically in vitro (Figure 1C).

**Figure 2:**
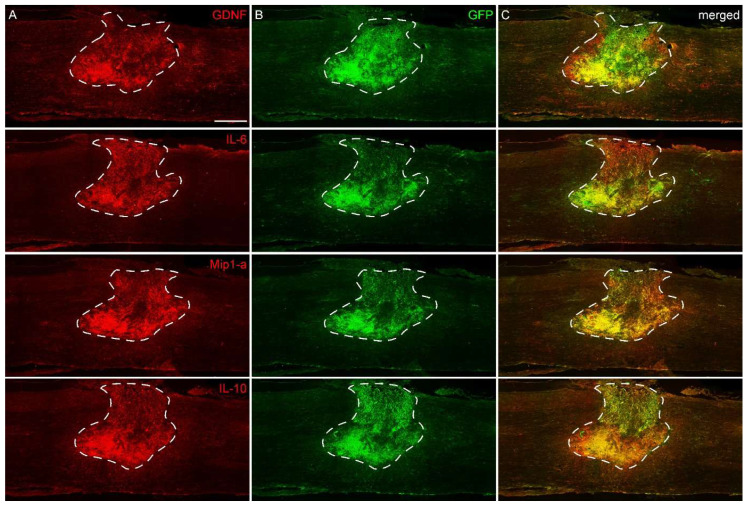
**Expression of four factors in grafted embryonic fibroblasts one week after transplantation.** (**A**,**B**) Representative images show the expression of all four factors and GFP in the injured spinal cord one week after grafting. (**C**) The embryonic fibroblasts expressing the factors colocalize with GFP. Note the expression pattern restricted to the borders of the graft. The broken line indicates the graft–host interface. Scale bar in (**A**): 500 µm.

**Figure 3:**
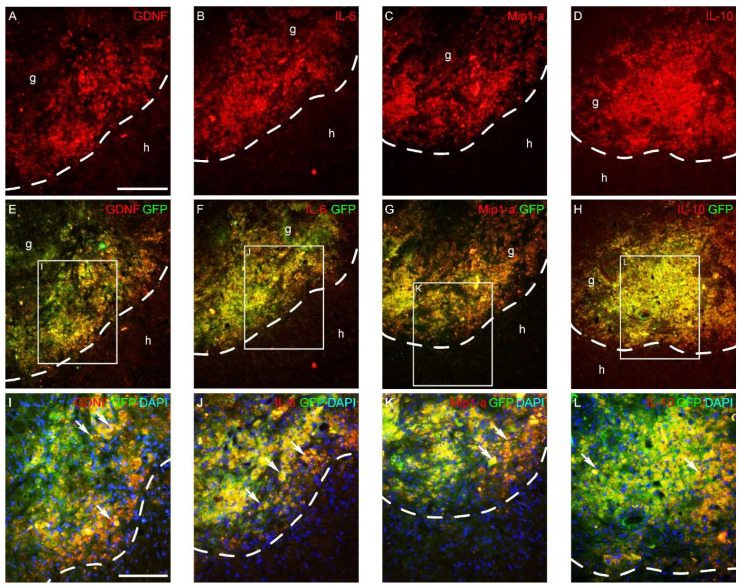
**High-resolution visualization of the four-factor expression in grafted embryonic fibroblasts one week after transplantation.** (**A**–**H**) High magnification of fluorescence images displays strong expression of GDNF, IL-6, IL-10, MIP-1a and GFP in the grafted cells. (**I**–**L**) High-resolution images of the boxed areas show that the vast majority of the GFP-positive grafted cells also express the factors. Arrows show co-localized cells in (**I**–**L**). The broken line indicates the graft–host interface. g: graft, h: host, Scale bar in (**A**): 200 µm, in (**I**): 100 µm.

Next, we investigated the in vivo expression of the four factors after transplantation. The transfected fibroblasts were grafted one week after injury into the lesion cavity, and expression of all four factors was analyzed with immunohistochemistry (Figure 2 and Figure 3). Immunofluorescence analysis revealed that graft-derived GFP-positive cells within the spinal cord expressed both cytokines (IL-6, MIP-1α, IL-10) and the neurotrophic factor GDNF. Notably, the expression of all four factors was localized predominantly in the graft (Figure 3). The factors were equally and simultaneously expressed one week after grafting.

### 3.2. Dose-Dependent Effect of IL-10 on Functional Recovery

To determine the dose of the factors to be delivered to the site of lesion (i.e., into the contusion cavity) we set up a dose-response curve of IL-10 administered to the injured cord via osmotic pumps. Based upon the literature and our previous data, it was expected that all four factors are acting with the same efficacy when applied in the dose of IL-10 [6]. Administration of various doses of IL-10 (7.5 to 45 ng/day) produced a dose-dependent increasing functional recovery of the treated animals with little difference between doses of 22.5 and 45 ng/day of IL-10 (Supplementary Figure S1). Therefore, we decided to use 22.5 ng/day of each factor in these experiments.

### 3.3. Lesion-Induced Secretome-Based Therapy Promotes Functional Recovery

**Figure 4:**
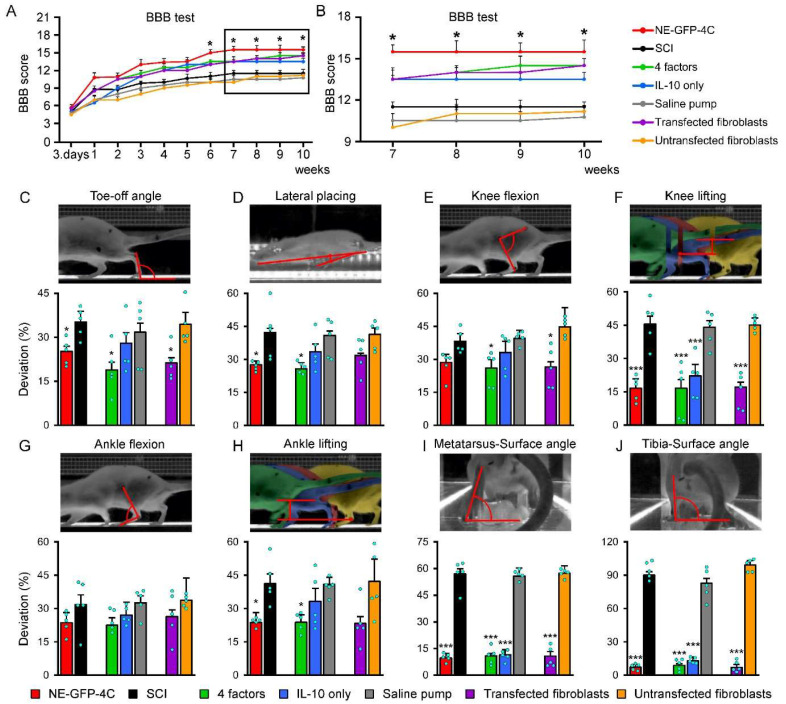
**Evaluation of functional recovery.** (**A**,**B**) Functional recovery was assessed by a weekly BBB test for 10 weeks after SCI. The pharmacological treatments induced significant recovery of locomotor function following SCI. (**C**–**J**) Detailed kinematic analysis of hind limbs from lateral and (**C**–**H**) caudal view (**I**,**J**). Note the significant locomotor pattern differences between the treated and control groups (*n* = 5 in each group) regarding most of the parameters. The treatment groups did not display significant differences between each other, suggesting the equal effect of the various treatment strategies. The values of the y axis indicate the deviation from the intact values. * (*p* < 0.05) and *** (*p* < 0.001) indicate significant differences between treated animals (Groups 1, 3, 4 and 6) and their control groups (Groups 2, 5 and 7). Data are presented as mean ± SEM.

Next, the functional improvement induced by the various therapeutical approaches was studied. For functional analysis, we used a BBB test and a detailed kinematic lower limb locomotor analysis. All the three pharmacological treatment groups [four factors (Group 3), IL-10 only (Group 4) and transfected fibroblast therapy (Group 6)] produced significantly higher BBB scores compared with their controls [~16 vs. ~11 points; SCI (Group 2), saline control (Group 5) and untransfected fibroblast (Group 7)]. The grafted NE-GFP-4C cells (Grp. 1) seemingly improved the general locomotor pattern to a slightly better extent in the BBB test than the secretome-based administrations (Figure 4A,B). The treated animals showed both consistent fore and hind limb coordination and weight-supported plantar stepping.

A detailed kinematic analysis of treated and control rats was performed at the end of survival time. Toe-off angle, lateral placing, knee flexion, knee lifting, ankle flexion and ankle lifting were analyzed in a lateral view and metatarsus-surface and tibia-surface angles in rear view (Figure 4C–J). No differential improvement between the stem-cell-grafted (Group 1) and secretome-based therapies [four factors (Group 3) and transfected fibroblast (Group 6)] was confirmed by the detailed locomotor test, where both therapeutic approaches showed equally prominent improvement compared with their controls (Figure 4C,F,I,J).

### 3.4. Delivery of Lesion-Induced Secretome Induces Tissue Sparing

**Figure 5:**
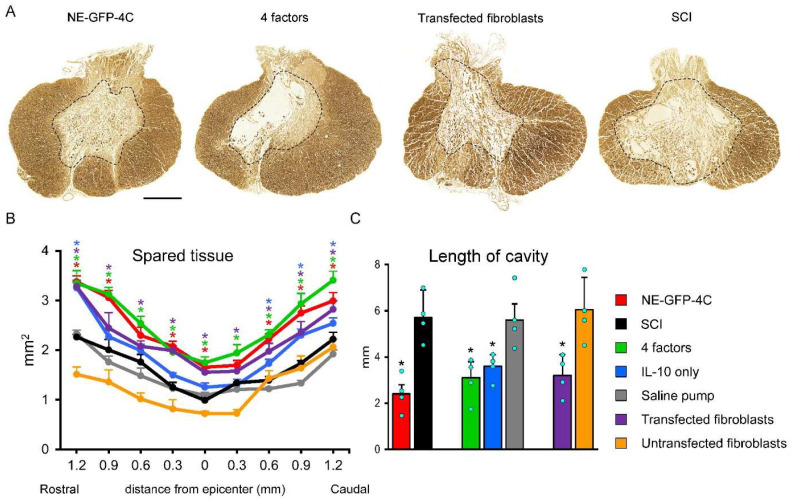
**The lesion-induced secretome treatment induces tissue sparing.** (**A**) Representative images taken from the lesion epicenter show observable differences between the treatment groups and the native solvent negative control group (SCI). Dashed line indicates the border between the intact and lesioned spinal cord regions. (**B**) Quantitative analysis of the spinal cord at different interval distances rostrally and caudally from the injury epicenter. IL-10 treatment alone appears to be less effective in tissue sparing at the epicenter compared to the other three treatment groups. (**C**) Bar chart diagram presenting the length of cavity measured in each experimental group. Data are presented as mean ± SEM. * (*p* < 0.05) indicates significant differences between treated groups (Groups 1, 3, 4 and 6) and their controls (Groups 2, 5 and 7). Scale bar: 500 µm in (**A**).

The functional improvement was paralleled by the morphological features of the contusion cavity. The extent of damaged tissue in the lesion area appeared to be remarkably smaller in the treated groups (Figure 5A). Quantitative measurements have clearly shown that the spared tissue was significantly larger in the stem cell or secretome-treated groups [NE-GFP-4C Group 1, four factors (Group 3) and transfected fibroblast (Group 6)] compared with their control groups [(SCI (Group 2), saline control (Group 5) and untransfected fibroblast (Group 7); Figure 5B].

A similar result was observed concerning the length of the cavity. By applying the factors, the length of the cavity was significantly reduced compared with control animals (Figure 5C).

Comparing the efficacy of IL-10 administration alone (Group 4) with that of all the four factors administered through the use of osmotic pumps (Group 3), NE-GFP-4C cell grafting (Group 1) and transfected fibroblast grafting (Group 6) revealed that IL-10 by itself was not able to achieve the same effect as in the other three groups.

### 3.5. Lesion-Induced Secretome Treatment Leads to Increased Number of Labeled Projection Neurons Rostral to the Injury After Retrograde Tracing

**Figure 6:**
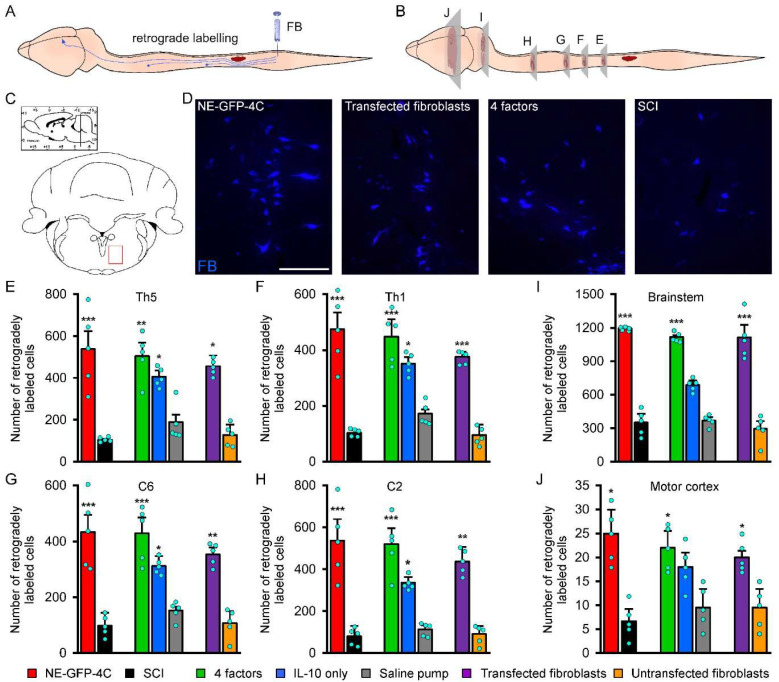
**Neuronal tracing analysis in various parts of the central nervous system.** (**A**–**C**) The schematic image shows the retrograde labeling procedure and the individual sampling locations. FB crystals were placed into the right hemisection gap of the L3 spinal segment. (**D**) Retrogradely labeled neurons are seen in pontine reticular formation of the brainstem. (**E**–**H**) Bar charts show the number of retrogradely labeled neurons at various levels of the spinal cord. IL-10 treatment (IL-10 only group) results in a significantly higher number of retrogradely labeled neurons compared to the control (saline pump group). (**I**,**J**) In the brainstem and motor cortex, significantly higher retrogradely labeled neurons can be found in the NE-GFP-4C, four factors and transfected fibroblasts groups. Data are presented as mean ± SEM. *, ** and *** indicate significant differences between treated groups (Gr. 1, 3, 4 and 6) with their control groups (Gr. 2, 5 and 7). * *p* < 0.05, ** *p* < 0.01, *** *p* < 0.001. Data are presented as mean ± SEM.

The propriospinal pathways play a crucial role in mediating reflex control and coordination during locomotion [28]. The extent of regenerating and spared spinal axons of various tracts was examined by retrograde labeling from the right L3 hemisegment of the injured cord (Figure 6A). Neurons retrogradely labeled with the fluorescent Fast Blue tracer were mapped and counted in the C2, C6, Th1 and Th5 spinal segments, brainstem, and motor cortex (Figure 6B–D). Both the stem-cell-grafted (Group 1) and the secretome-treated animals (Groups 3 and 6) had significantly more retrogradely labeled cells in all investigated areas, indicating far better proprio/supraspinal control of the spinal cord segments caudal to the injury in the treated animals compared with their controls (Figure 6E–J). IL-10 treatment alone (Group 4) significantly augmented the number of retrogradely labeled cells in the above areas, but to a lesser extent as compared with the other treatment study groups (Figure 6E–J).

### 3.6. Corticospinal Tract Sparing/Regeneration Induced by Lesion-Induced Secretome Treatment

**Figure 7:**
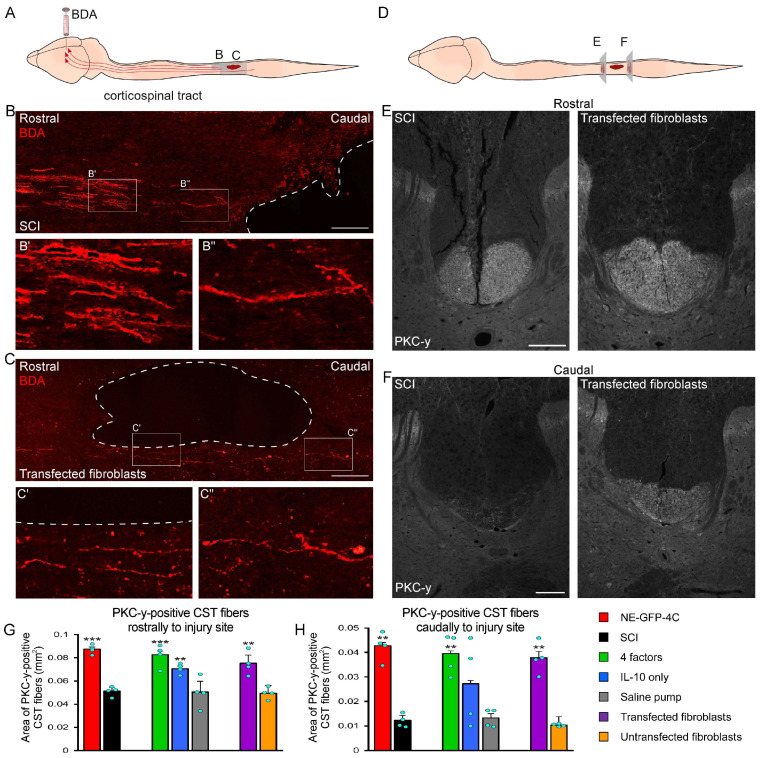
**Secretome-based therapy promotes growth of dCST axons caudal to the injury.** (**A**) Schematic image shows the anterograde labeling procedure. BDA was injected into the motor cortex. (**B**,**C**) Low magnification parasagittal images encompassing the lesion site at 12 weeks after SCI are shown. Labeled corticospinal fibers are apparent ventrally and caudally to the lesion in animals treated with transfected fibroblast, but not in the control group (SCI). (**B’**,**B”**,**C’**,**C”**) Higher-magnification views demonstrate the BDA-positive fibers. (**D**) Schematic image shows the source of the images presented in (**E**,**F**). (**E**,**F**) Cross sections taken 2 mm rostrally and caudally to the ends of the lesion show PKCγ-positive fibers of the dCST at 12 weeks after SCI. (**G**,**H**) There was a significantly higher area of PKCγ-positive dCST fibers rostral and caudal to the injury in NE-GFP-4C, four factors and transfected fibroblasts group compared to controls (SCI, saline pump and untransfected fibroblasts). Data are presented as mean ± SEM. ** *p* < 0.01, *** *p* < 0.001 indicate significant differences between treated groups (Groups 1, 3, 4 and 6) with their control groups (Groups 2, 5 and 7). Dashed line indicates the border between the intact and lesioned spinal cord regions. Scale bar: 200 µm in (**B**), 50 µm in (**C**), 200 µm in (**E**,**F**).

The spinal cord contusion injury completely interrupts the dorsal corticospinal tract (dCST) in rats [28]. The sparing/regeneration of dCST was examined by labeling the motor cortex of the animals with biotinylated dextran amine (BDA; 10 kDa) injections. BDA was taken up by large pyramidal cells and transported along the whole length of their axons anterogradely into the spinal cord (Figure 7A). The anterogradely labeled axons of the dCST were mapped around the lesion site. In the case of the secretome-treated animals (Groups 3, 4 and 6), sparing/regenerating corticospinal axons approached the very cranial pole of the lesion cavity and then turned ventrally to grow along the ventral wall of the contusion cavity (Figure 7C). Many of the BDA-positive fibers reached the caudal end of the cavity where they returned to their original pathway within the base of the dorsal funiculus. On the other side, the anterogradely labeled corticospinal axons approached, but did not reach the rostral border of the lesion cavity in the control animals (Figure 7B). In order to quantify the amount of sparing/regenerating dCST fibers, protein kinase C-γ (PKCγ) immunohistochemistry was performed. PKCγ is a marker used to detect and follow changes of plasticity of certain CNS tracts, especially that of dCST [17]. Indeed, rostrally to the cranial pole of the injury cavity (2 mm), the tract was outlined in a uniform and clearly visible manner both in control (Groups 2, 5 and 7) and treated animals (Groups 1, 3, 4 and 6) with an observable difference in favor of the treated cords (Figure 7D–F). However, caudal to the injury (2 mm), PKCγ-positive fibers were barely detectable in the control groups (for reference, only Group 2 animal images are shown), while a well identifiable PKCγ-positive area could be observed in the treated groups (for reference, images only from Group 6 animals are shown, Figure 7D–F).

Quantification of the PKCγ-positive areas rostrally and caudally to the injury in each experimental group are shown in Figure 7G,H. A significantly larger PKCγ-positive area was identified both rostrally and caudally in the spinal cords of treated animals (Groups 1, 3 and 6). Analysis at both sampling sites (cranially and caudally to the cavity) showed that NE-GFP-4C-grafted animals displayed the largest PKCγ-positive area, but this value turned out to be non-significantly different from that of the other four-factor treatment groups (Groups 3 and 6). It is important to note that IL-10 treatment alone (Group 4) resulted in a remarkably larger PKCγ-positive area but it was not significantly different compared with its control (Group 5). However, in animals treated with the four factors (Group 3 and 6) or stem cells (Group 1), the PKCγ-positive area was nearly 30% greater than that of the IL-10 only group.

### 3.7. Modulation Effect of the Lesion-Induced Secretome Treatment on the Lesion Microenvironment

**Figure 8:**
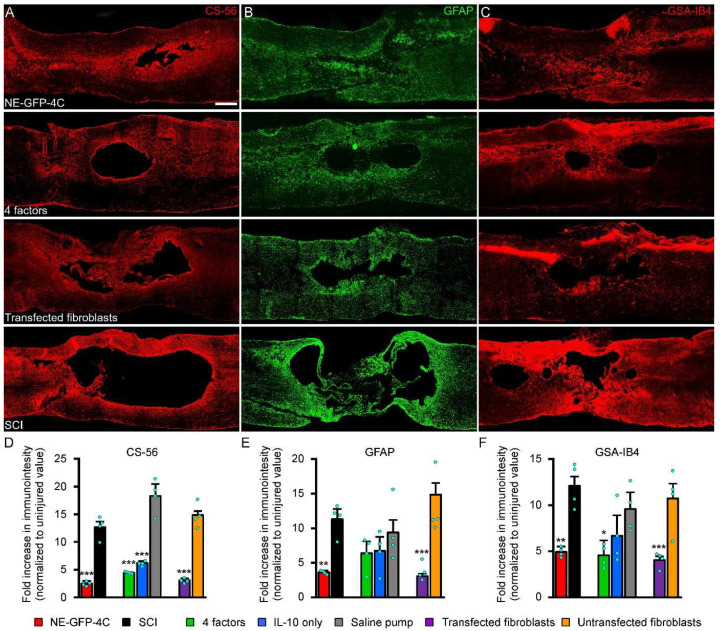
**Effect of the lesion-induced secretome treatment on glia reaction around the lesion.** (**A**–**C**) Representative images of parasagittal sections show the CS-56, GFAP and GSA-IB4 isolectin reactivity 10 weeks after the injury. (**D**–**F**) Quantification of CS-56, GFAP and GSA-IB4 isolectin density in the various experimental groups. All the pharmacological treatments decrease the CS-56 and GSA-IB4 density. Treatment via osmotic pump is not able to influence the astroglia reaction around the lesion site. Data are presented as mean ± SEM. * *p* < 0.05, ** *p* < 0.01, *** *p* < 0.001 indicate significant differences between treated groups (Groups 1, 3, 4 and 6) with their control groups (Groups 2, 5 and 7). R = rostral, C = caudal; Scale bar: 500 µm.

To detect the effects of the various treatment strategies on the glial changes and deposition of extracellular chondroitin sulphate—a major regeneration-inhibiting molecule—we determined the levels of chondroitin sulphate deposits, astro- and microgliosis by using the markers CS56, GFAP and GSA-IB4 ten weeks after injury, respectively (Figure 8A–C). All the three relevant markers displayed significant downregulation due to the treatment effects. Both the cellular therapy (NE-GFP-4C grafting, Group 1) and the secretome factors delivered by osmotic pumps (Group 3) or produced by transfected fibroblasts (Group 6) prevented the severe deposition of chondroitin sulphate (Figure 8A). On the other hand, both NE-GFP-4C stem cell grafting (Group 1) and administration of transfected fibroblasts (Group 6) successfully downregulated astro- and microgliosis in the injured cord, while the delivery of the secretome factors by osmotic pumps (Group 3) was effective only in reducing microgliosis (Figure 8C). Interestingly, IL-10 treatment alone (Group 4) had some observable, but non-significant, effects on the glial response.

### 3.8. Lesion-Induced Secretome Treatment Protects the Myelinated Axons Following SCI

**Figure 9:**
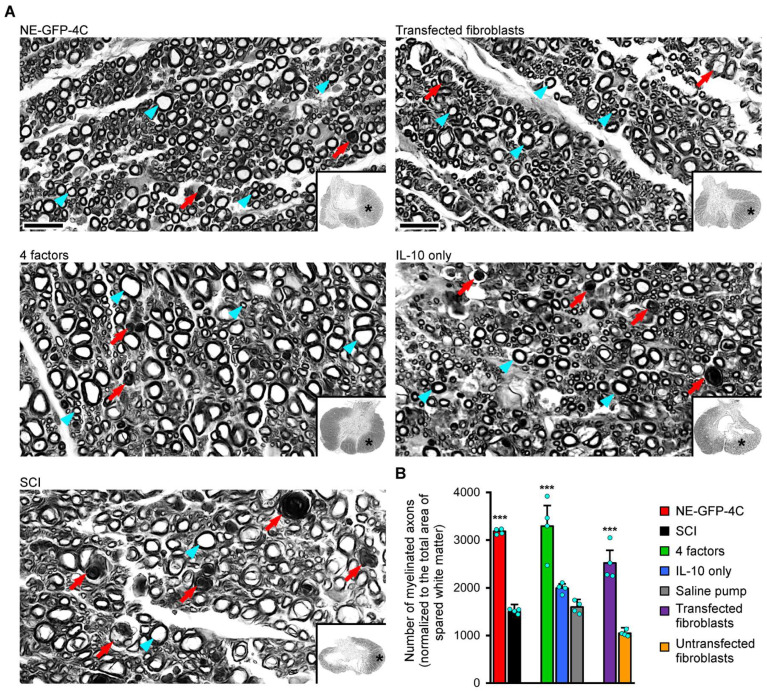
**The lesion-induced secretome protects myelinated axons.** (**A**) Photographs of semithin cross-sections from the epicenter of the lesion 10 weeks after injury. The stem-cell-treated (NE-GFP-4C group) or the lesion-induced secretome treated spinal cords (four factors and transfected fibroblasts group) contain more myelinated axons (turquoise arrowheads), while the IL-10 treated (IL-10 only group) or control spinal cords display far fewer regenerated/spared myelinated axons (turquoise arrowheads). Red arrows show abnormal myelin sheaths. Asterisks indicate locations of higher magnification view. (**B**) The chart shows the numbers of myelinated axons found in the lesion epicenter 10 weeks after the injury. IL-10 treatment alone is not able to protect myelinated axons. Data are presented as mean ± SEM. *** indicates significant differences between treated groups (Groups 1, 3, and 6) to their control groups (Groups 2, 5 and 7). * *p* < 0.001; Scale bar: 50 µm in (**A**).

The number of myelinated axons remaining intact after a spinal cord lesion is critical for the functional outcome of the injury. To determine the number of myelinated axons at the level of the epicenter, the tissue blocks of epicenter regions were paraffin-embedded and osmicated (Figure 9A). The number of myelinated axons was determined and normalized to the total area of the spared white matter in 5 μm thick transverse sections. Three treatment strategies (Groups 1, 3 and 6) resulted in significantly higher numbers of myelinated axons as compared with their controls (Groups 2, 5 and 7), except for the IL-10 only treatment (Group 4), which did not produce significant improvements in the sparing of myelinated axons (Figure 9B). Accordingly, successful treatments yielded a minimum of twice as many myelinated axons as were found in the control spinal cords.

## 4. Discussion

Here we have demonstrated the long-term benefits of a delayed stem cell secretome-based therapy in terms of neuroprotection, neuronal regeneration and restoration of motor function after low-level thoracic spinal cord contusion injury. Remarkably, various drug delivery methods of the secretome (GDNF, IL-6, IL-10 and MIP-1a) all promoted tissue sparing and regeneration of the CST and other fiber tracts accompanied by improvement in several functional measures of locomotion and weight support with trunk stability.

Our previous study demonstrated that delayed transplantation of NE-GFP-4C neuroectodermal stem cells into the spinal cord injury area promoted significant tissue sparing and locomotor recovery due to the lesion-induced secretome produced by the grafted stem cells [6]. In the above study, the underlying mechanism was a paracrine effect that significantly contributed to the neuroprotection and neuronal regeneration through decreased glia reaction and trophic support.

In the present study, we have provided evidence that different drug delivery methods of intralesional secretome administration equally reduced the total lesion area and increased the amount of the spared white matter—including the number of myelinated fibers—and fostered the regeneration of injured axons. It is important to highlight that increased tissue preservation and fiber tract regeneration correlate with improved motor function recovery. The secretome-based therapies (NE-GFP-4C grafts, four-factor delivery via osmotic pump, and transfected fibroblasts) not only improved functional recovery, but were also closely associated with the preservation of myelinated axons, attenuation of glial reactivity, and regeneration of the corticospinal tract (CST), and likely that of other spinal cord pathways. Sparing of myelinated axons and remyelination of regenerated neurites are of particular importance, as both axonal conduction velocity and long-term axonal survival critically depend on intact myelin sheaths; accordingly, the significantly higher number of myelinated fibers observed in the treated groups strongly correlated with improved motor outcomes. This observation is consistent with previous reports demonstrating that remyelination is a crucial part of functional recovery after SCI [29,30]. In parallel, modulation of the glial response emerged as another key factor. The reduction in astrocytic (GFAP-positive) and microglial activation, together with decreased chondroitin sulphate deposition, indicates the establishment of a more permissive extracellular environment for the axonal regeneration. This is in accordance with other studies showing that reactive gliosis and glial scar formation represent major inhibitory barriers to axonal regeneration, while their attenuation promotes axonal sprouting and plasticity [31,32]. Importantly, our data support the concept that fine-tuned modulation rather than complete suppression of glial activity is optimal for regeneration.

The preservation and partial regeneration of the corticospinal tract is of particular importance, as it represents the anatomical substrate of voluntary motor control in humans. Anterograde tracing with BDA and PKCγ immunohistochemistry revealed significantly increased CST sparing/regeneration, both rostral and caudal to the lesion site in treated animals. This is particularly noteworthy, as CST regeneration in adult mammals is very limited and typically requires combinatorial therapeutic strategies [33,34]. Our findings therefore suggest that the multi-component secretome not only provides neuroprotection but also creates a microenvironment that supports long-distance axonal growth/sparing and pathway reorganization. Compared to single-molecule approaches such as IL-10, the four-factor secretome appears to have a broader and more effective regenerative effect, consistent with studies highlighting the benefits of secretome therapies [13,35].

Some components of the secretome (such as GDNF and IL-10) are well known to induce significant axon regeneration and anti-inflammatory effects by acting along different signaling pathways [36,37,38,39]. GDNF is a well-known neurotrophic factor that can promote the growth of axons and the survival of the injured tissue through its receptor, GFRalpha [40]. IL-10 is a widely known, potent anti-inflammatory cytokine, which binds to its receptor (IL-10R) and activates the JAK/STAT signaling pathway. Due to this, expression of pro-inflammatory cytokines (TNF-alpha, IL-1B, IL-12, INF-gamma) decreases in the target cells. On the other hand, IL-10 promotes anti-apoptotic processes enhancing the survival of injured cells [10,41,42]. These effects were clearly represented by the results gained through the use of a single IL-10 (IL-10 only) treatment of the injured cord. IL-10 treatment induced a powerful improvement in the modulation of the lesion microenvironment (downregulation of astrocyte and microglia reactivity and chondroitin sulphate deposition) and tissue sparing. Considerable improvement was achieved in the number of regenerating CST fibers, while retrograde labeling showed a more consistent sparing/regenerative effect, and a limited effect was observed in the (re)myelination of axons traversing through the lesion site. These findings have significance in future clinical applications of IL-10 as this cytokine appears to be most easily administered in human therapeutic approaches. Therefore the single use of IL-10 in this experimental approach is justified from the clinical therapeutic point of view, too.

The other two components of the secretome (IL-6 and MIP-1a) are better known for their pro-inflammatory roles in the peripheral immune system. IL-6 can act as both a pro- and anti-inflammatory cytokine [43,44,45]. Although its inflammatory role is better known, numerous studies have provided evidence that IL-6 is also able to induce neuroprotection in the nervous system through IL-6R-mediated STAT3/ROS regulation and inhibiting neuronal apoptosis and inflammatory mediators [46,47,48].

MIP-1a is a pro-inflammatory cytokine/chemokine, which is connected to its receptor (CCR-1, CCR-3, CCR-5) via the Gq protein by promoting the activation of the JAK/STAT signaling pathway and contributing to the enhanced inflammatory processes [49,50]. The role of MIP-1a is not well understood in this scenario, but its modulating effect has already been demonstrated in our earlier studies [5,6,11].

Our results have clearly shown that all four factors are required for the promotion of the complete regenerative and restorative processes. Indeed, IL-10 therapy alone via osmotic pump proved to be successful in providing neuroprotection, but did not foster the long axonal regeneration and sparing without the other three factors. Therefore, it appears that the harmonized action of the four factors together is needed to produce a powerful neuroprotective and regenerative effect in the injured cord.

Secretome-based therapy represents a plausible strategy to harness neuroprotection/neuroregeneration following SCI [12]. Over the years, experimental secretome-based therapies have greatly contributed to the repair and regeneration of damaged tissues after SCI [16,51,52]. As confirmed by several studies, secretome-based drug delivery supports the axonal remyelination and regeneration of the host, and contributes to the overall protection and preservation of tissues [17,53,54,55,56]. In addition, these therapies have anti-inflammatory, anti-scarring, anti-apoptotic and proangiogenic properties, which greatly enhance functional recovery after SCI [35,57,58,59].

The composition of the secretome produced by various stem cells, independent of their origin, shows quite a high degree of variability. These stem cell secretomes consist of a number of proteins such as hepatocyte growth factor (HGF), vascular endothelial growth factor (VEGF), and TGF-β1, IL-10, NT-3, FGF, etc. [60,61]. The different protein compositions of the secretomes can synergistically exert an effect on the damaged environment and prevent secondary damage [62]. However, characterizing the composition of the secretome reveals significantly different secretory profiles of stem cells, which appear to vary depending on the experimental conditions used; therefore, it is important to determine the secretome composition of a lesion, as this composition largely determines its effectiveness.

Taken together, these data indicate that secretome-based therapy derived from very efficient stem cells establishes a favorable environment for regenerating axons of the already-rescued neurons after SCI. In line with these results, we have demonstrated in our experiments that the secretome can induce a high degree of tissue protection by expressing it through an osmotic pump or by using pVAX-SB transfected fibroblasts. It is important to emphasize that the treatment in our experiments involved an effective secretome treatment for a period of 7–10 days, which is absolutely necessary to achieve therapeutic effects. Based on our results and other literature data, it seems that the therapeutic application of the secretome by various drug delivery ways represents a viable alternative to stem cell transplantation in the treatment of SCI.

## 5. Conclusions

It can be concluded that the neuroprotective and regenerative efficacy of the NE-GFP-4C stem cell line shown in spinal cord contusion injury can be reproduced to the same extent through the use of the stem cell secretome via various drug delivery routes as with intraspinal grafting of stem cells. This finding opens new avenues in the use of effective stem cell secretomes for the treatment of CNS disorders and injuries.

## Data Availability

All data for submission is available on the following link: https://doi.org/10.5281/zenodo.19236349.

## Supplementary Materials

The following supporting information can be downloaded at: https://www.mdpi.com/article/10.3390/pharmaceutics18060658/s1, Supplementary Figure S1. Dose dependent effect of IL-10. Functional recovery was assessed by BBB test weekly for 9 weeks after SCI. The IL-10 (22.5 ng and 45 ng group) treatments induced significant recovery of the locomotor function following SCI compared with control animals (SCI group). Supplementary Figure S2. Transfected fibroblasts express all four factors. Transfected fibroblasts display strong expression of GDNF, IL-6, IL-10 and MIP-1a co-localized with GFP 1 week after transfection. Scale bar: 20 µm.

## Abbreviations

The following abbreviations are used in this manuscript:

SCI	Spinal cord injury
NE-GFP-4C	Neuroectodermal stem cells
GDNF	Glial cell line-derived neurotrophic factor
IL-6	Interleukin-6
IL-10	Interleukin-10
MIP-1 alpha	Macrophage inflammatory protein-1 alpha
GFP	Green fluorescent protein
PBS	Phosphate-buffered saline
PFA	Phosphate-buffered paraformaldehyde
pVAX-SB	pVAX-based Sleeping Beauty polycistronic vector
TOA	Toe-off angle
KF	Knee flexion
AF	Ankle flexion
KL	Knee lifting
AL	Ankle lifting
LP	Lateral placing
MSA	Metatarsus-surface angle
TSA	Tibia-surface angle
FB	Fast Blue
BDA	Biotinylated dextran amine
GSAI-B4	Griffonia Simplicifolia isolectin B4
DAPI	4′,6-diamidino-2-phenylindole
PKCγ	Protein kinase C-γ
GFAP	Glial fibrillary acidic protein
CS56	Chondroitin sulphate-56
CST	Cortico-spinal tract
GFRalpha	GDNF family receptor alpha
JAK/STAT	Janus kinases/signal transducers and activators of transcription
TNF-alpha	Tumor necrosis factor alpha
IL-1B	Interleukin-1beta
IL-12	Interleukin-12
INF-gamma	Interferon gamma
IL-6R	Interleukin-6 receptor
STAT3/ROS	Signal transducer and activator of transcription 3/reactive oxygen species
CCR-1	C-C chemokine receptor type 1
CCR-3	C-C chemokine receptor type 3
CCR-5	C-C chemokine receptor type 5
Gq protein	Guanine nucleotide-binding protein subunit alpha q
HGF	Hepatocyte growth factor
VEGF	Vascular endothelial growth factor
TGF-β1	Transforming growth factor-beta 1
NT-3	Neurotrophin-3
FGF	Fibroblast growth factor
CNS	Central nervous system
