# Supplementary material for: Fostering Regeneration and Functional Improvement in the Injured Spinal Cord by a Novel, Stem Cell Secretome-Based Drug Delivery Method
Source: Pharmaceutics. 2026 May 27;18(6):658. doi: 10.3390/pharmaceutics18060658 (PMC13305432; doi:10.3390/pharmaceutics18060658)
Supplement: Supplementary file 1 [file pharmaceutics-18-00658-s001.zip › pharmaceutics-4291152-supplementary.pdf]

Supplementary Materials

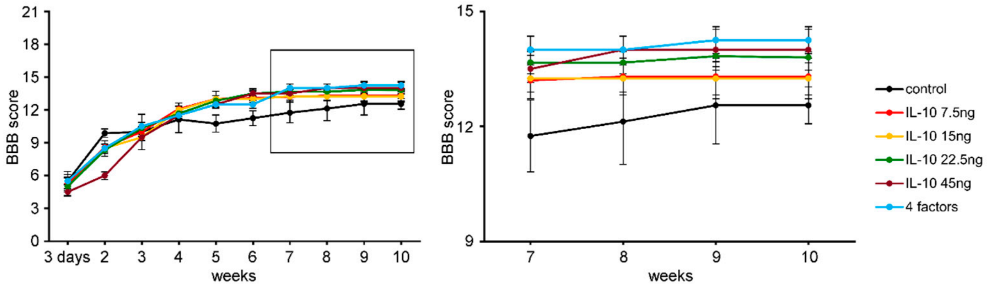

Figure S1. Dose dependent effect of IL-10. Functional recovery was assessed by BBB test weekly for 9 weeks after SCI. The IL-10 (22.5 ng and 45 ng group) treatments induced significant recovery of the locomotor function following SCI compared with control animals (SCI group).

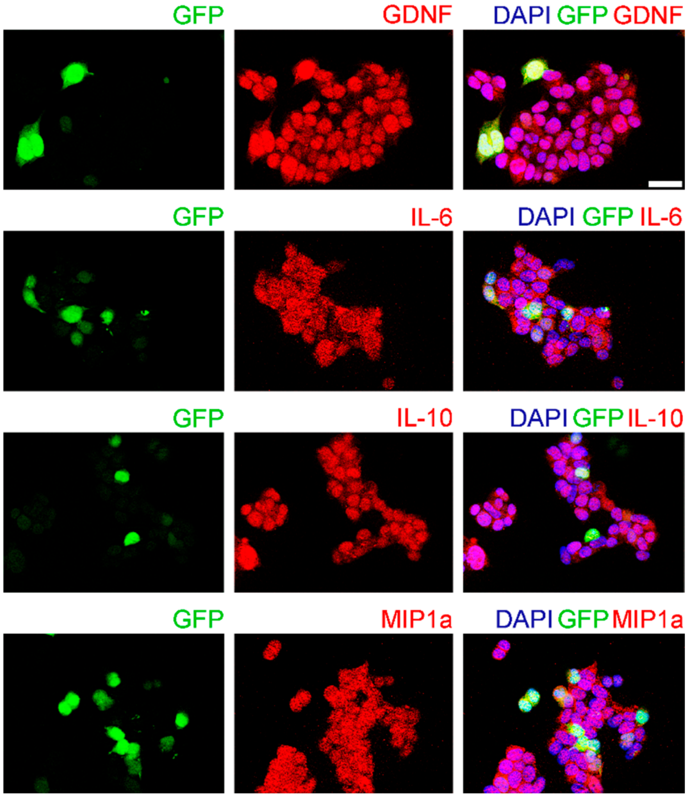

Figure S2. Transfected fibroblasts express all four factors. Transfected fibroblasts display strong expression of GDNF, IL-6, IL-10 and MIP-1a co-localized with GFP 1 week after transfection. Scale bar: 20 µm.
